# M. Dupuytren's Clinical Lectures at the Hôtel Dieu

**Published:** 1833-07-01

**Authors:** 


**LEÇONS ORALES DE CLINIQUE CHIRURGICALE, FAITES A L'HÔTEL DIEU DE PARIS.** Par *M. le Baron Dupuytren*, Chirurgien en Chef. Recueillies et publiées par une Societé de Médecins. Tom. 1 et 2. A Paris, 1832. (Clinical Lectures on Surgery, delivered at the Hotel Dieu, in Paris, by Baron Dupuytren.)

[Continued from page 321 of Vol. XVIII.]

### ART. XV. ON LUXATION OF THE VERTEBRÆ, AND ON DISEASES WHICH ARE LIABLE TO BE MISTAKEN FOR IT.

CASE 1.—*Laceration of the Ligaments of the Bodies of the Vertebræ without displacement.* A man, æt. 50, was standing firmly at the tail of a cart, with his head and neck bent forward, whilst a side of beef was being placed on his back, when the burden, slipping from the hands of the man who held it, fell with force on the neck of the butcher, and threw him on the ground. He was brought into the Hôtel Dieu. There was a large ecchymosis without any tumour at the posterior and inferior part of his neck, which was painful when touched or moved. At the same point a distinct crepitation was heard when the head of the patient was raised or turned. There was loss of motion and feeling in the arms and legs, in the walls of the thorax and abdomen, with paralysis of the bladder, and retention of urine. The muscles of the diaphragm, face, and neck, were alone capable of contraction; respiration was difficult. He remained two or three days in this state; respiration then became more embarrassed, and he died from suffocation. On examination a large ecchymosis was found around the last cervical vertebræ. The intervertebral substance uniting the fifth and sixth was completely destroyed, but the bodies of these vertebræ were uninjured. The spinous, transverse, and articular processes of the fifth, sixth, and seventh cervical vertebræ were broken, so that the two portions of the spinal column could be readily displaced. The medulla spinalis at the part appeared rather more voluminous but otherwise healthy; however, on making a longitudinal section, the centre was found to be almost putrid and mixed with decomposed blood.

CASE 2.—*Laceration of the Ligaments of the Bodies of the Vertebræ, with Displacement.* A labourer, between 40 and 45 years of age, employed at the quarries, whilst his body was bent forwards, received a mass of earth on his loins, the weight of which, after some efforts on his part to overcome it, threw him on the ground. He was taken home, where he remained three days with complete loss of sensation and motion in the inferior parts of his body. On the fourth day he was brought into the Hôtel Dieu. At the upper part of the loins was a large tumour, hard in the centre, and soft in the circumference, where evident crepitation was felt. Another tumour was felt through the abdomen in the track of the spine. The length of the abdomen was much diminished, and the base of the chest almost touched the cristæ of the iliac bones. There was complete loss of sensation and motion in the lower limbs, and abdominal parietes. Urine flowed involuntarily from the distended and paralysed bladder. Costiveness; belly distended, but soft; pulse small and contracted; respiration short and difficult. Dull pain in the lumbar region; intellectual faculties unaffected. The tumours, crepitation, diminution in the space between the chest and pelvis, and the paralysis, clearly indicated a solution of continuity of the spinal column with displacement. The paralysis gradually increased, and on the seventh day the patient died asphyxiated. On examination it was found that the transverse and articular processes of the last dorsal vertebra, and of the two first lumbar, were broken. The body of the last dorsal vertebra, and that of the first lumbar, separated from their articulations, and from the body of the second lumbar vertebra, had passed before this latter bone, projecting above an inch. The spinal marrow was lacerated, and the pillars of the diaphragm torn. On carefully examining the displaced vertebræ it was found that their intervertebral substance had been lacerated, and that a thin layer of bone had been also torn from one corner of the lumbar vertebra.

Case 3. A tall and large woman, 56 years old, in descending a staircase at night, fell backwards, and struck violently against the edge of a step the posterior and inferior part of her neck. On raising her it was found that nearly every part beneath her neck was deprived of sense and motion. In the morning she was brought to the Hôtel Dieu, suffering acute pain at the lower part of the neck, increased by the slightest touch or motion. The head and neck were inclined forwards, and a little to the right, the posterior part of the neck was depressed, and the superior part of the dorsal region elevated. Complete paralysis beneath the diaphragm, partial paralysis of the arms, respiration frequent and laborious, voice and intellectual functions natural. From these symptoms it was evident that there was solution of continuity of the spine, with displacement. The following night the respiration became more difficult, and she died.

*Examination of the Body.* The cellular tissue and muscles adjacent to the injury were bathed in blood. The parts immediately in contact with the vertebral column were destroyed, so as to expose the superior articular processes of the seventh cervical vertebra, whilst the sixth was driven half an inch before the seventh. In the interval between these two bones the medulla spinalis was seen stretched from behind forwards, and from above downwards, flattened, and compressed on the body of the seventh cervical vertebra. On examining the spine anteriorly, an eminence surrounded by blood was evident, consisting of the whole of the thickness of the body of the sixth cervical vertebra. The anterior ligaments of the spine were destroyed, and the pharynx torn. On detaching the cervical portion it was found that the ligamenta subflava, the anterior and posterior vertebral ligament, as well as the intervertebral substance uniting the sixth and seventh vertebræ, were torn; that the seventh cervical vertebra was entire in all its parts; that the apex of the spinous process, as well as the borders of the inferior articular processes, were slightly bruised. The articulations of the vertebræ above the luxation were natural; that between the seventh cervical and the first dorsal was much more moveable than natural.

The first of these cases is an example of laceration of all the ligaments of the vertebra, and fracture of their articulating processes without displacement of the bones; in the second, these same injuries are attended with displacement; the third is a pure and simple luxation without any previous fracture. In all three cases the spine has been in a state of tension, and the force has been applied behind. In all three cases has the intervertebral substance been torn; in all, the injury of the medulla spinalis was the cause of the dangerous nature of the injury, and of the immediate paralysis. In the three cases, the paralysis has been gradually propagated from below upwards, until, the origin of the diaphragmatic nerves having been implicated, the patient has died from interruption of the various phenomena of respiration. From some of the observations of M. Dupuytren, it appears that the possibility of luxation of the vertebræ, under any circumstances, is doubted by many French surgeons. In this country some are still inclined to doubt the possibility of luxation without fracture, and to such this case of the Baron's, in which he confesses that the summit of the spinous processes and the articular processes were "légèrement entramés," would not be allowed to be a decisive proof to the contrary. However, more than one instance of luxation of the cervical vertebræ without fracture are recorded by our own countrymen, and it is a point which can be decided by one well-authenticated case as well as by a thousand, and in a practical point of view is of little value. Its extreme rarity all must admit. The diagnosis of these injuries is explained by cases.

CASE 1.—*Rheumatism simulating Luxation of the Cervical Vertebræ.* A boy, aged 15, was brought to M. Dupuytren by several practitioners, who believed that he had dislocated his neck. Two days previously, in endeavouring to take off his shirt, he made a violent movement, and at the same time felt acute pain in the cervical region, and heard a crack, his head remained firmly bent towards the left side. The surgeons who were called in, pronounced it an incomplete luxation of the first vertebra on the second, but as the case appeared to them obscure and dangerous, they requested M. Dupuytren's opinion. On examination he found the head strongly bent to the left side, and the spinous processes of the first vertebra projecting; the neck was rounded at the opposite side; he felt great pain at the part, which was increased on the slightest effort made to rectify the displacement of the head. There was numbness and pricking pains in the right shoulder and arm; the patient swallowed with difficulty, and was unable to turn or bend his head. M. Dupuytren immediately decided that there was no luxation, but that the affection was rheumatic. He had often seen in persons subject to rheumatism, the occurrence of acute pain in the neck after some effort, owing to the erratic rheumatism becoming fixed. Thus persons whilst dancing are suddenly seized with acute pain in the calf, which hinders them from walking for two or three days, and which does not at all depend on rupture of the tendon of the plantaris; others, in mounting into a cabriolet, feel suddenly so acute a pain in the common mass of the sacro-lumbales and longissimi dorsi muscles, that they step back alarmed, believing they have been struck or wounded. Such pains, when they cease in one part, not uncommonly attack another. M. Dupuytren was inclined to attribute this cause to the accident, as the patient was employed in a wine-shop, and was obliged frequently to descend into the cellar and to remain there a considerable time daily; he had also had rheumatic pains. Blood to be taken by cupping from the left side of his neck. In the morning the correctness of the diagnosis was proved by the amelioration of the symptoms. Emollient fomentations, &c. were employed, and in a fortnight the patient left the hospital, having lost every trace of the affection.

Case 2.—*Distention and Swelling of the Cervical Intervertebral Ligaments.* A man was admitted into the Hôtel Dieu on account of pain, &c. in the cervical region. Some months previously he had fallen down, and subsequently he stated that he had been struck across the neck and head with a gun. There was change in the form of the neck—it was concave anteriorly, convex posteriorly, with projection of the upper cervical vertebræ. He was unable to rotate his head; and when he wished to turn himself, in order to look or walk, the head appeared soldered to the trunk. The disease was aggravated by work, and swelling of the cervical ligaments had supervened. Cupping, and the subsequent application of two moxas, produced much relief. The pain subsided, and he could move his head with ease.

The following case will be perused with interest, as it not only illustrates the functions of the nerves of the tongue, but tends to prove the truth of Sir C. Bell's discoveries.

A man, aged 30, was admitted last year into M. Dupuytren's wards. He had been a weaver, a trade which subjects those who follow it to rheumatic affections, as they work either in cellars, or in damp and cold ground-floors. Three years previously, he was seized with acute pains in the left side and back part of the head; these pains prevented all motion of the part, and deprived him of rest. In five or six days, the pain shifted to the upper part of the left side of the neck. The lateral flexion of the head, as well as the motions backwards and forwards, were partially executed by the whole cervical column, but not by the two first vertebræ. These symptoms were followed by slight difficulty in speaking; this increased, so that, at the end of two months, the patient could not make himself understood. He said that, in whistling, the air passed by the left side of his tongue, and that when he wished to say *je,* he pronounced *ze.* There was pain in the angle of the left lower jaw, but no paralysis of its muscles. The left side of the tongue then began to diminish in size, and the anterior and middle part became so completely atrophied, that it felt like an empty leather purse. The mucous membrane was entire, but the muscles beneath it had disappeared. The right side was well nourished, and appeared to have gained an increase in strength. For some months, at first, the patient could not articulate, but when examined, he could enunciate clearly and distinctly. He regained his speech by practice with the maimed organ, rather than by any diminution of the disease. M. Dupuytren ascertained, by experiment, that the patient had not lost the sensation of taste in the atrophied half of the tongue. The atrophied parts are, therefore, the muscles, and the consideration of the distribution of the nerves of the tongue to these muscles led M. Dupuytren to form his diagnosis. The tongue is more abundantly supplied with nerves than any part of the same size; they are the hypoglossal, glosso-pharyngeal and lingual branch of the fifth pair. The lingual branch of the fifth is distributed to the papillæ on the upper surface of the tongue; if this had been affected, the taste would have suffered, the glosso-pharyngeal supplies the posterior part of the tongue and the lateral parts of the pharynx, and the freedom of these from disease proves that the nerve that supplied them was not implicated. The great hypoglossal nerve, or ninth pair, which arises between the corpora olivaria and pyramidalia of the medulla oblongata, escapes from the cranium through the anterior condyloid foramen. It furnishes branches to the muscles inserted in the os hyoides, and to the middle cervical plexus, and terminates in the muscles of the tongue, of which it is the motor nerve. M. Dupuytren considered that this was the nerve affected. The freedom from any disturbance in the intellectual faculties, as well as in the organs of motion, proved that the disease of this nerve was not within the cranium, but after its exit from that cavity. This opinion is strengthened by the disease of the occipito-vertebral articulation. The impossibility of executing the motions of the head seemed to indicate an affection of the ligaments uniting the vertebræ, probably arising from rheumatism; the situation of the pain in the first instance shewed that the disease existed between the atlas and the occipital bone, perhaps also between the atlas and dentata. The left side of the neck, which, at its upper part, is supplied by the ninth pair of nerves, was slightly atrophied. As the hypoglossal nerve passes through a foramen, in front of the occipital condyle, it was probably either compressed or disorganized by the inflammatory engorgement of the ligaments uniting the atlas with the os occipitis. The treatment adopted was energetic, as there were symptoms threatening an extension of the disease to the right side. Blood was repeatedly drawn by cupping from behind the mastoid processes, and moxas were subsequently applied. These remedies were successful.

Anchylosis of the atlas and os occipitis are not uncommon; there are 10 or 12 preparations of this affection in the Museum of Comparative Anatomy; but M. Dupuytren has never seen another case of paralysis and atrophy of one-half of the tongue.

### XVI. ON BURNS.

To this subject M. Dupuytren has devoted particular attention, and he has frequently been in the habit of giving a series of clinical lectures upon it. The division of these injuries most commonly adopted is founded on the symptoms, thus three degrees are generally acknowledged; first, simple inflammation ending in resolution; second, in suppuration; and the third, complete destruction of the vitality of the part. M. Dupuytren's divisions depend on the tissues that are implicated. They are six. 1. Erythema, or simple phlogosis of the skin without vesications. 2. Cutaneous inflammation, with separation of the epidermis by vesication. 3. Destruction of the rete mucosum, and papillary surface of the dermis. 4. Disorganization of the whole dermis as deep as the subcutaneous cellular tissue. 5. Eschars of the superficial parts, and of the muscles wholly or partially. 6. Carbonization of the whole thickness of the burned part.

1. This is usually produced by radiant heat, hot vapours, &c. The erysipelatous redness is attended with smarting pain, these symptoms disappear in a few days, and the cuticle desquamates. If a large surface is implicated, symptoms of gastro-intestinal irritation supervene. If the skin of the head is burned, the irritation may be propagated to the brain.

2. This is the effect of a more energetic cause, of longer continuance. The pain is sharp and burning, and at the same time, or most frequently some hours afterwards, vesications appear; if these, after a few days, are broken, the rete mucosum, covered with a new epidermis, still thin and reddish, is seen. In some cases the epidermis is at once destroyed; this is followed by intense pain and slight suppuration.

3. This is marked by grey, yellow, or brown spots, insensible if slightly touched, but painful if pressed. The vesications covering them are filled generally with a brownish, milky serosity, or bloody serum, and this is at first a useful diagnostic mark. The detachment of the eschar leaves superficial ulcerations, the cicatrices of which remain almost always conspicuous, owing to the white, dense, and shining layer which replaces the part destroyed. When the inflammation necessary to the detachment of the eschar commences, the pain, which after the first 24 hours was trifling, returns violently. It should be remembered, that the pain produced by burns is much greater when the surface of the skin only is burned, than when it is deeply destroyed.

4. If an ignited body has remained some considerable time on the part, it causes acute pain which ceases on the cause being removed. The epidermis, rete mucosum, the whole thickness of the dermis, and sometimes a slight layer of subcutaneous cellular tissue are deprived of life, and reduced to a deep, yellowish, or blackish, dry, insensible eschar. The healthy skin around it is puckered and shrivelled by the heat. In three or four days the pain commences, an inflammatory circle forms around the eschar, which in 15 or 20 days is separated; the bottom of the wound is formed by subcutaneous cellular tissue, which supplies a very abundant suppuration, and vigorous granulations. The force with which the circumference of ulcerations from burns is drawn towards the centre is a phenomenon almost peculiar to these injuries; it is not observed in any other wounds with loss of substance in any comparable degree; hence deformities are so common a result. These results never take place on the posterior part of the trunk, as the natural flexion of the body opposes the contraction of the cicatrix; the same observation applies to the limbs.

5. In this degree the eschars comprehend aponeurosis, muscles, and tendons. The consequent suppuration is much more abundant than in the other cases, and the cicatrix, as the motor organs themselves are implicated, is imperfect, adherent, and productive of irremediable injury to the motion of the part.

6. The surface of the limb is carbonized, hard, insensible, sonorous on percussion, and easily broken if it is attempted to be bent; the eschar, when detached, leaves an irregular stump. MM. Roche and Sanson have published a case of a young man who, stepping into the trench in a foundry through which a stream of melted iron was running, was overtaken by the red hot fluid, and who drew out the limb with the loss of his foot and the lower part of the leg. He hardly felt pain, and did not perceive at first the horrible mutilation.

Such is M. Dupuytren's division, which differs from the other in its more accurate distinction of the injuries to the skin, &c. which had been comprehended by authors under their last head. But it must not be imagined that cases will often occur answering solely to either of these degrees. In extensive burns every degree may be recognized on different regions of the body. In many cases these divisions are not easily recognized immediately after the accident. The action of the fire which has disorganized the more superficial parts, often has so much injured those immediately subjacent, that they cannot withstand the inflammatory action, and consequently lose their vitality. Thus eschars, when detached, expose a greater loss of substance than was at first imagined; this is important in a medico-legal point of view, as in burns of the third and following degrees no opinion should be given until the eschars begin to separate. Constitutional symptoms are either the immediate results of general irritation produced by the action of caloric, or the consequences of subsequent inflammation. The pain produced by the action of a concentrated heat on the animal solids may be so intense as to cause immediate death. M. Dupuytren has seen several of these cases of "death by excess of pain," and he believes that an excessive loss of sensibility may kill, as well as an excessive loss of blood in hæmorrhages. Children and nervous women are most subject to such a termination, adults rarely, and old people never. There are symptoms of violent irritation of the cerebral system, and of congestion of almost all the organs of the great cavities. In other cases, where the irritation is not sufficiently intense to be immediately fatal, there are frequently, excessive agitation, loss of sleep, spasms, convulsions, fever, or the patients fall into a state of collapse, which usually terminates fatally in a short time; in a few cases there is a general re-action.

In superficial burns of some little extent there is a constitutional re-action similar to that in erysipelas, which readily yields to medical treatment. Irritable patients are particularly subject to it. In many burns of the third and fourth degrees there are no unfavourable symptoms until about the fourth day, when inflammation commences. Symptoms of nervous and gastric irritation then set in with greater severity than in the more superficial injury, and sometimes terminate fatally. There is often great oppression and difficulty in breathing, owing to the severe shock which the organs of respiration and of articulation have undergone, and to a secondary bronchial irritation, or pulmonary congestion. Excessive and protracted suppuration gradually exhausts the strength, produces emaciation, and incurable marasmus. One of the most severe and dangerous complications is phlegmonous erysipelas; even when its progress is arrested, the disorganization it has effected is so excessive, that amputation affords the sole, but doubtful prospect of success. Thus the constitutional effects of burns may produce a fatal issue in four ways; 1st, by irritation; 2dly, inflammation; 3dly, suppuration; 4thly, exhaustion. In the examination of the bodies of those who have perished in a general conflagration, either in the midst of the flames, or in a few minutes after having been dragged out of them, there are marks of considerable congestion of the digestive canal. The mucous membrane not only presents bright red patches of various sizes, but is injected with blood, and contains some of this fluid in its cavity which has escaped by exhalation. The brain is strongly injected, there is a reddish serum in the ventricles, as well as in the cavities of the pleura, pericardium, and peritoneum. The bronchial tubes contain a bloody mucosity, their mucous membrane is in many points of a bright red, or covered with injected capillaries. It appears as if the blood, driven from the surface by a sudden and general irritation, had endeavoured, under the influence of the excessive stimulus of the heart and vascular system, to traverse the free pores of the internal surfaces. If life is prolonged two or three days, and death takes place from inflammatory re-action, preceded by symptoms of acute visceral irritation, all the usual effects of acute gastro-enteritis are observed, accompanied usually by inflammatory appearances of the brain and of the lungs. The effects of inflammation of the lungs, unsuspected during life, such as Stoll has described as latent, are often discovered. If life has been protracted until the patient has been worn out with suppuration and exhaustion, the viscera, and particularly the digestive canal, presents all the appearances produced by long-continued inflammation. The mucous membrane is covered with red patches, or in a state of ulceration, &c.

*Prognosis.* Extensive burns of the first degree may produce death immediately, or in a few hours after the injury; but when 24 or 48 hours have elapsed, resolution commences, and the danger is passed. When the burning body is very hot, and its application rapid, it may produce a kind of tumefaction of the epidermis; if the surface thus affected is very large there is great danger. This accident sometimes is produced by a bath which is too hot. In burns of the second degree the same effects may be produced, but besides these, there is more danger of inflammation of the internal organs, and the patient is not in safety from such an attack until desiccation has commenced. It should be borne in mind, that in all cases women, children, and irritable, nervous patients, are less able to bear the pain consequent on burns, than adults and old people. In burns of the third degree, there is not only a liability to all the effects of the first two, but to those which may arise from the establishment of the inflammation necessary to detach the eschars. If a considerable surface is affected, such as two or three feet square, death generally takes place when the secondary inflammation or suppuration are established. In burns of the fourth and following degrees the irritation and pain usually remain no longer than the actual cause, but the sufferers may perish during this period. If they survive it, in some instances they are plunged into a state of stupor, they are icy cold, and die in a few hours; or they rally, and are cut off from the fifth to the ninth day by inflammatory re-action, or finally the profuse suppuration, the length of the disease, hospital gangrene, or a low fever produce fatal exhaustion.

*Treatment.* In burns of the first or of the second degree, which are not attended with destruction of the epidermis, the indications are, to prevent inflammation, vesication, and the formation of eschars. Slightly astringent and sedative applications, which do not stimulate, fulfil this indication. Such are, long-continued immersion of the part in cold water, or in goulard water, or in water slightly accidulated, or mixed with a little alcohol; when immersion is impracticable, long-continued and frequently-renewed fomentations with the same liquids, or with ether, alcohol, a solution of sulphate of iron, alum, or ammonia. These latter substances cannot be employed except the epidermis be uninjured, for if it be removed they augment the irritation and produce acute pain. It is therefore important to preserve the epidermis, and for this purpose great care must be taken in removing the clothes. If vesications are formed they must be opened with a needle at their most depending part. Sedatives or topical anodynes, if the pain is considerable. Local or general bleeding if the subject is young, vigorous, and sanguineous. Diet restricted in proportion to the severity of the injury. If, notwithstanding these precautions, inflammation commences, it must be attempted to be moderated by emollient fomentations and cataplasms, local and general bleedings. In burns of the third or fourth degrees, the same treatment is indicated when the secondary inflammation commences. This should be restrained, if too violent, and excited under opposite circumstances; but it should be recollected, that too energetic or long-continued stimulants may produce erysipelas, which is sometimes fatal. M. Dupuytren, in such cases, applies a temporary blister to the erysipelatous surface, which almost always puts a stop to the inflammation. At this period the burn should be covered with fine linen, having numerous small holes cut in it, and smeared with simple cerate or saturnine ointment; above this should be placed a slight layer of charpie, in order to absorb the pus. Emollient cataplasms should be applied to the sloughs to detach them, and when they are almost entirely loosened, the few filaments which confine them to the bottom of the wound should be cut with scissors. Where the slough is deep, and a feeling of fluctuation indicates the presence of pus, an incision should be made through the slough to give the fluid exit, and to prevent its infiltration into the surrounding cellular tissue. When, after the separation of very superficial eschars, or of the epidermis after vesications, the exposed dermis is very painful, an opiate cerate should be applied, or the dressings should be moistened with a weak solution of the gummy extract of opium. In dressing a burn, it should be exposed as short a time as possible to the air, to prevent pain; a many-tailed bandage is, therefore, preferable, when it can be applied, as, by that means, a part of the wound only need be exposed at a time. After extensive burns of the third or fourth degree, suppuration is generally very abundant, so that it becomes necessary to renew the dressings two or three times daily. Substantial food and tonics, such as quinine, given by the mouth, in lavements, and applied topically, are necessary. The deformities resulting from malformed cicatrices may almost always be prevented, by carefully cauterizing the too prominent granulations, by position, by well-applied dressings, and the use of firm splints.

When a limb, or part of a limb, is completely destroyed, amputation is indispensable; it substitutes a simple wound, which will readily cicatrize, for an irregular solution of continuity, with projection of the bone, as well as the secondary inflammation. The age, constitution, and strength of the patient must of course be taken into account.

We have entered thus fully into M. Dupuytren's observations, not from a conviction of the utility, in general, of minute divisions, nor of any novelty in his treatment, but from a persuasion that a promulgation of such views, founded on pathological principles, is the best method of removing the false idea which is attached, even by many of the profession, to the word burn. How is it that every age has had its specific—that each has fallen in its turn into disrepute, and yet that infallible remedies still continue to be discovered? Is it not owing to the simple idea which is attached to the word—*burn?*

Now it is M. Dupuytren's object, by dividing these accidents according to the texture implicated, to prove that a burn is not a simple, but a very compound disease, consisting of many and various degrees, presenting decided characteristic marks, differing in the effects they produce and in the treatment they require; this treatment is to be based on the same general principles which actuate the practice of the surgeon in similar injuries, produced by other causes. It would not be more absurd to class destruction of the skin, rupture of a muscle, ecchymosis, and fracture of a bone, under the head blow, and recommend a single application for its cure, than to comprehend under the same term the complicated injuries of destructive burns, and recommend turpentine, wool, or flour, as an infallible specific in all.

### Art. I. Vol. II.—Cicatrization of Burns.

Where there has been actual loss of substance, a new structure, of a peculiar fibro-cellular texture, is formed, which M. Dupuytren denominates "tissue of cicatrix" ("tissu de cicatrice.") If the work of re-production proceeds regularly, the loss of substance is in great measure repaired; but, under opposite circumstances, a bad cicatrix is formed, producing deformity, adhesions, &c. These results may follow any loss of substance, but they vary according to its depth and extent.

1*st Degree.* When this is followed by erythema, as there is no loss of substance, there can be no cicatrix; but in chronic cases, as in women who use chaufferettes, and old people or invalids, who sit very near the fire, &c. the long-continued application of heat produces stains, and even destruction of the epidermis.

2*d Degree.* In the mildest vesications, as in blisters, which are removed as soon as a vesication is formed, a new epidermis is produced in from one to five days, and redness of the part only remains, which disappears in a few weeks. Such an application produces no change in the colour of the negro's skin. But if the action of heat has been more intense, or that of a blister prolonged, a serous discharge takes place for a few days, followed by suppuration; this may be mild, or, if neglected, may be abundant. The effects of profuse suppuration may be a complete destruction of the rete mucosum, as in the third degree, or partial disorganization, in which case the colouring matter becomes of a much deeper hue, and the scars are marked indelibly with yellow or brownish spots. In the negro, the skin of such cicatrices is of a darker colour than that of the surrounding parts. Where the rete mucosum has been destroyed completely in some parts, and disorganized in others, the cicatrix is variegated. The knowledge of these facts will be useful in the application of blisters, as in women, or in conspicuous parts of men, the suppuration should not be continued long enough to produce a scar. In cases where the long-continued action of a blister is necessary, it is better to apply numerous ones for a short time, sometimes on one place, sometimes on another. It is clear that, in these consequences of burns, when the inflammation passes its ordinary limit, it should be diminished by emollients, &c. and even by bleeding, care being taken to avoid topical irritants, and to remove the broken epidermis, in order to hinder or suppress the suppuration. In those cases where the surgeon is called in after tedious suppuration has destroyed the rete mucosum, or disorganized it, he must equalize the granulations, by rubbing the surface of the wound with nitrate of silver, and dress it with compresses of linen, covered with ointment; employ desiccating substances, and sheet lead to produce equal pressure. These means generally produce an even cicatrix, but they will not remedy the yellow or brown discolorations; indeed time very rarely even modifies their colour.

*3d Degree.* In this, the epidermis and rete mucosum are completely destroyed, together with a portion of the true skin, and the cicatrix is formed by means of the layer of the corium which remains. When the eschar is detached, numerous red points are seen at the bottom of the wound, on a whitish ground; this is formed by part of the corium which has not been destroyed, and the red points are the summit of the small cellular bundles, fixed in the areolar cavities, and supporting the nerves, arteries, veins, and lymphatics, which are distributed on the external surface of the corium. These red points soon increase in number, and so totally obscure the whitish ground, that the wound has a uniformly red appearance. The cicatrix is, therefore, formed by these cellular, vascular, and nervous elevations. But, however instantaneous has been the application of the heat, it never destroys the tissues to an equal depth; hence the inequality of the granulations, and, if neglected, the uneven cicatrix. In the treatment, similar means to those recommended in the second degree are necessary; adhesions between separate parts are to be prevented by the interposition of foreign bodies, as linen smeared with cerate, charpie, &c. and the obliteration of natural openings opposed by the introduction of tents, canulæ, &c. M. Dupuytren has used for many years, with great success, an ivory instrument, so constructed as to be introduced into the nostrils where a portion of the nose has been destroyed by a burn, or removed in an operation. It is of great importance to apply the dressings so that the pus may be immediately absorbed, and remain as little as possible in contact with the wound. For this purpose, M. Dupuytren applies immediately over the burn a fold of linen, pierced with numerous holes, very close to each other, and above that a layer of charpie, confining the whole with a many-tailed bandage. After suppuration has continued some time, the granulations become more firm and fibrous, this change commencing at their base. A thin epidermis is then deposited on their surface, under which the rete mucosum is formed, of a bright red colour, irritable, easily thrown into a state of congestion, and often the seat of erysipelas. This imperfect rete mucosum is generally deprived of pigment. Thus, in the negro, such a cicatrix is white, and whiter in the European than the surrounding skin. In other cases, it is wholly or partially re-produced; but such a new production is always imperfect—its colour is not similar to the old one, nor can this be remedied. The epidermis, at first thin and scaly, acquires in time all the properties, except the colour, of the old one.

*4th Degree, or complete Destruction of the Corium.* After the eschar has sloughed, the bottom of the wound formed by the subcutaneous cellular tissue is red and irregular, from the numerous granulations which cover it; in its edges is seen a red circle, the rete mucosum, beneath which a white circle, or the corium; these layers are unequally destroyed. The wound contracts daily, its swollen edges subside and approach each other, the surrounding skin yielding to the traction. If the wound is very large, the skin having arrived at its maximum of extension and displacement, a new tissue replaces that destroyed. If the skin or position allows sufficient extension, the edges unite. When, in order to prevent deformity, a position is adopted which opposes the approach of the edges of the wound, it is repaired by a new structure. Thus, if the skin of the occiput is burned to the fourth degree, the cellular tissue being short, and not readily extended, a new tissue is formed; if, on the other hand, the skin is destroyed on the palm of the hand, and of the palmar surface of the fingers, and the case is neglected, the edges of the wound will contract as far as possible, until the fingers are immoveably fixed together, or to the palm; but if the hand has been forcibly extended, a new tissue is produced, and the motions of the hand are free. Similar deformity takes place in burns of the axilla, ham, neck, &c., or the deformity is reversed if the burn is in the direction of extension, thus, in burns of the back of the hand, the fingers may be bent backwards, &c. The following is the mode in which the cicatrix is formed. The granulations, which are at first small, are in process of time completely changed, their cellular substance becomes cellulo-fibrous, and very hard; this replaces the corium; when once this is formed the new skin is speedily completed, a very imperfect rete-mucosum, deprived of pigment, is produced, and an epidermis, which in a short time differs very little from the natural membrane. The production of the new corium is a very difficult process, (or, according to the present French fashion of *explaining* such difficulties, "la production coûte beaucoup a la nature"!) but as soon as this is effected, the remainder of the cicatrix is completed with remarkable rapidity. Thus it is often astonishing to see a wound which has continued one, two, or more months without any change, heal suddenly in a few days.

In the treatment of the cicatrization of these wounds, the first remedy is position of the limb, and the general rule to regulate this is, to place the part in a position diametrically opposite to that which will favour the cicatrization of the wound by the approach of its edges. The end to be obtained by this is to form a cicatrix of the size of the skin which has been destroyed, and even of greater dimensions, as the new tissue is peculiarly retractile. Thus, if the fold of the elbow-joint, the palm of the hand, the groin, ham, &c. are burned, the limbs must be forcibly retained extended until the cicatrix has formed, and vice versâ. But where the skin of the whole circumference of a limb is destroyed, a medium position must be preserved, so that the cicatrix may be of as little inconvenience as possible; or the limb placed sometimes in one position, sometimes in another, in order to hasten the cicatrization on one side, and to retard it on another. In some destructive burns the weak state of the patient prevents position being attended to, as the necessary precautions would endanger life. In such cases the friends should be informed of the inevitable consequences. In some regions of the body the regulation of position is impossible, or extremely difficult, as in the face. Thus in a burn of the fourth degree, implicating the lower eye-lid and the cheek, it is impossible to prevent the junction of the edges of the wound, so that the eye-lid is almost united with the upper lip. Great deformity also results from burns of the forehead, temples, upper eye-lid, hairy scalp, &c. When position cannot thus be employed, the fall of the eschar should be delayed as long as possible, as this tends to separate the edges, and when it has fallen, the cicatrization must be hastened by frequently cauterizing the part with nitrate of silver. M. Dupuytren has found that this application hastens the formation of an accidental cutaneous tissue, rendering the cicatrix equal, flat, and exempt from those deplorable elevations which expose the want of skill or negligence of the surgeon. The wound should be frequently washed and dressed. Position should be persevered in for a month, six weeks, or more, after the cure of the patient, and then should be gradually suspended; for the contractile power of the new tissue may produce much subsequent retraction. Bandages are necessary to retain the parts in position. Thus, if the anterior, posterior, or lateral parts of the neck are burned, in order to bend the head in the proper direction, leather straps should be fixed by some turns of a circular bandage to the head, and by their other ends to a bandage round the body. If the anterior part of the arm is burned, extension should be kept up by a posterior splint and circular bandage. If any part of the circumference of the wrist is burned, a pad is to be placed on the opposite side of the arm, reaching to the wrist, above that a splint extending to the ends of the fingers, and taking advantage of the space between the hand and the splint, the former is to be bent on the latter by some few turns of the bandage which was employed to fix the pad. If the palm of the hand and fingers are burned, the extension is to be kept up by means of a pad on the posterior surface, and over that a splint the size of the extended hand, having ten holes for little bandages to confine the fingers, or cut into the shape of fingers. In burns of the second and third degrees, adhesions of contiguous surfaces may be prevented by separating them by the interposition of foreign bodies, and moving them at each dressing; but in more severe burns compression must be employed, by a long and narrow strip of linen, the middle of which is to be placed in the angles formed by the junction of the fingers, and the ends are to be fixed to the fore-arm, one in front and the other behind it; if this is not attended to, the hand will have the appearance of the foot of a web-footed animal. Where either of the natural openings of the body are thus burned, great attention must be paid to the constant introduction of tents, canulæ, &c. which should always exceed in size that of the natural outlet, and should be continued for a long time after the cure. Adhesive plasters may be of some use in preventing deformity when position cannot be employed.

*5th Degree.* In these burns, where the motor organs are so deeply involved, nothing else can be done than by placing the part in such a position as will produce the least inconvenient cicatrix; but generally the disorganization is so great, that in order to save life, cicatrization must be accelerated without regard to subsequent deformity.

*Physical, Anatomical, and Pathological Characters of the Cicatrices succeeding Burns.* It is not until many weeks or months after a wound is healed that the cicatrix is completely formed, it gradually acquires a density and thickness necessary to supply the deficient skin, and at the same time diminishes in extent. This retraction does not cease until the cicatrix has become white and solid. If examined in this state it will be found to be covered by a thin, shining, and very adherent epidermis, beneath which is a dense tissue formed of fibrous layers crossed in every direction; a substance analogous to the corium; no trace of a rete mucosum remains, and consequently the cicatrix of a Black is similar in colour to that of a White. This new product contains neither sebaceous follicles, nor hairs, that is, if the whole thickness of the skin has been destroyed; the cicatrix is consequently dry, and the absence of the sebaceous fluid should be supplied during the progress of retraction by oily applications, warm and gelatinous baths, &c. It is pierced with very few exhalants or absorbents, hence it is dry, whilst the surrounding parts copiously perspire. A laminated tissue, more or less compact and deprived of fat, unites the cicatrix to the subjacent parts, and is depressed in proportion to the loss of substance which it has replaced, or to the quantity of surrounding adipose tissue. If muscles, tendons, &c. have been partially destroyed, the cicatrix adheres closely to them, and they lose for a short distance their natural texture, and degenerate into an homogeneous fibrous tissue, which is intimately united with the new product. A practised eye distinguishes immediately the cicatrix of a burn from that produced by scrophula, syphilis, &c.; in legal medicine this is of great importance, and no opportunities of examining such injuries should be lost. The vascularity of cicatrices is variable. Commonly there are but very few capillaries, into which it is very difficult to throw injections. From the sensibility of these parts when touched or inflamed, and in changes of weather, it is probable that they receive a few nerves. Like all anormal organized products they are destroyed by inflammation with prodigious rapidity. In a few hours or days the reparatory work of many months is destroyed; however, this distinction is often confined to the upper layer of the new product, which is speedily restored. They are generally unaffected by exanthematous diseases. Cutaneous adhesions uniting burned parts are at first large, soft, and reddish, becoming like the cicatrices more intimate and solid as their organization is completed. Subsequently they are elongated by the motion of the parts, decreasing in breadth as they increase in length, so that they are changed into thin membranous layers, their free border thickened and forming a kind of cord. Analogous at this stage to the membranes of web-footed animals, they are completely organized, and never change their form or size. Until they have attained such organic perfection they should not be subjected to surgical operations.

*The Means of correcting Deformities resulting from the vicious Cicatrices of Burns.* These consist either in too narrow or too prominent cicatrices, or in unnatural adhesions and obliterations. These are to be remedied only by a severe operation; which will be more easy of execution and successful in proportion as the adherence is superficial. If the motor organs are implicated the deformity may be corrected, but the motions of the part can very seldom be restored. An operation should not be attempted, 1st. until some months or even years have elapsed since the formation of the cicatrix, as the new substance is so imperfectly organized that it may be destroyed by the least cause, or even spontaneously. 2dly. Unless a larger cicatrix may be obtained by position or bandages; this applies especially to the face. 3dly. Unless it will restore the forms and functions of the parts. It will therefore be useless if the articulations are anchylosed, or the muscles destroyed, &c. The operation which M. Dupuytren has adopted is the following.

If it is required to remedy the deformity arising from a very narrow cicatrix, it is necessary, 1st, to make many incisions of the same length and depth as the cicatrix. 2d, To extend the parts, in order to bring them into a direction opposite to that into which they have been drawn by the cicatrix, and to obtain a new cutaneous tissue. If the parts are flexible, they may be brought immediately into the natural position; but if they are stiff, the extension must be slow and gradual, otherwise excessive pain, inflammation, &c. will result from the employment of force. A mechanical apparatus attached to the splints, by which a permanent and gradual extension is kept up, is of great utility. 3d, Subsequently to the operation, the same treatment is to be adopted as has been recommended after the detachment of the eschar of a burn. Secondary adhesions often form, which must be divided. If the cicatrix is too prominent—1st. A double-bladed, thin scalpel must be held flat, and passed through it about its middle; the cicatrix should then be removed on a level with the skin, by cutting out towards each extremity. 2d. The lips of the wound should be separated. 3d. The surface should be cauterized constantly, in order to keep the granulations a little below the level of the skin. If there are simple unnatural adhesions, 1st, they should be freely divided; 2d, the parts should be retained separate; 3d, a methodical compression on the angle of union should be kept up. If it is required to enlarge a contraction, or to remedy an obliteration of a natural opening, a knife or trochar should be used, and meshes or ivory tubes introduced. The new cicatrices have the same tendency to contract as the old ones, hence the necessity of long-continued extension; and baths, emollient applications, and oily embrocations.

In deformities produced by scars of burns of the second and third degree the operation will not be severe as they are very superficial. If adhesions exist between large surfaces, as between the arm and trunk, or the two thighs, the whole operation should not be attempted at once, as it might produce a dangerous wound. M. Dupuytren appeals to his clinical pupils for many years past to prove the success of his operations. Two cases are detailed, and a large body of facts are promised. The first is that of a child of four years old, whose little, middle, and ring-fingers of one hand were flexed on the palm, owing to a neglected cicatrix from a burn of that part. Transverse and perpendicular incisions were made, and the hand was extended for four months, when the parts had recovered their functions. In the second case, the hand of a child, 10 years old, was bent on the fore-arm by a membranous connexion extending from the middle and anterior part of the fore-arm to the palm of the hand. This resulted from a neglected burn eight years previously. This connexion was divided by three transverse incisions; gradual and constant mechanical extension was kept up for six weeks, when the child left the hospital, having a very fair use of his hand.

M. Dupuytren objects to the total removal of a cicatrix as likely to expose the patient to the most serious consequences, by laying bare subcutaneous cellular tissue, aponeuroses, and even bones, from which violent inflammation and profuse suppuration may ensue; and, also, as the disease may have an indefinite duration, and as in many cases it will be impossible to obtain an entirely new cicatrix to replace the lost tissue or the old scar. These objections are inconclusive as they are applied to an operation in cases in which the destruction is so great that Mr. Earle would hardly advise his operation or even M. Dupuytren recommend incisions. The success which has attended Mr. Earle's own cases fully prove the utility of his practice in less severe injuries, but still the severity of the operation, and the tedious cicatrization of the wound, are serious objections to its general adoption. It is to be hoped that this plan of M. Dupuytren's, which (although not acknowledged) is but a modification of Mr. Earle's, the principle of which was the formation of a new and more extended tissue in the place of the contracted one, as it offers less difficulty and danger, will be found to be attended with results as successful. Experience of a large number of cases which have stood the action of *time*, the great test of all these operations, can alone decide. We are sorry that the compilers of these lectures have not given a general table of the results of the numerous operations stated to have been performed at the Hôtel Dieu.

Fractures of the Neck of the Os Femoris. M. Dupuytren enters into a methodical and clear explanation of the causes of this injury, and of the changes which age produces in the form and structure of the neck of the femur and of the surrounding parts, rendering it an accident almost peculiar to advanced life. Into these details our space will not allow us to enter, and we shall proceed at once with the treatment. The common mode in France is to keep the whole limb in an extended position by means of Dessault's splint; the inconveniences of this unnatural position, and the little success which attends it, struck M. Dupuytren, and he has been in the habit of applying to the treatment of this injury the principle so strongly recommended by Pott in common fractures of the shaft of the thigh-bone, that of semiflexion of the limb. Although this may be a novel treatment in Paris, it is not so in this country. It is commonly adopted in some of those hospitals (St. Bartholomew's for instance) which are supplied by Mr. Earle's ingenious mechanical beds, and we ourselves have been in the habit of employing pillows for the same purpose on the recommendation of a judicious country surgeon who has adopted the plan for many years. The directions however which M. Dupuytren has given are of such easy application, and the advantages which he states to have resulted from their employment are so great, that we make no apology for their insertion, particularly as the treatment of fractures by simple apparatus, which is at every one's command, when thus recommended, is of first-rate practical importance. The experience in the treatment of some of these injuries which a student gains in a large hospital furnished with every useful mechanical contrivance, is often of little service when he is thrown on his own resources in general country practice, particularly among the poor, who are most subject to such injuries, and the greatest sufferers by insufficient treatment. By simply raising the thigh so as to bend it on the belly, and using moderate traction, the leg being semiflexed and the pelvis fixed by assistants, reduction is accomplished, the limb regaining its natural length, and the foot its usual position. This is owing to the relaxation of the adductor muscles which had turned the foot outwards, and of the glutei and others which had drawn upwards the inferior fragment. If then the demiflexed position is the best to reduce the fracture, and to maintain it reduced, it follows that the best apparatus is that which retains the muscles semiflexed; this is the principle laid down by Pott, but singularly it was not applied by him to fractures of the cervix femoris. M. Dupuytren forms his double-inclined plane with pillows. A pillow rolled into the cylindrical form of a bolster and retained in this shape by tapes, is to be placed at the top of two inclined planes, formed by many pillows placed one above the other, and united by sewing them together by one of their borders. One of these oblique planes extends from the ham to the tuber ischii, the other from the ham to the heel, their apex being at the angle of union of the thigh and leg. The thigh is to be fixed by a cloth folded " en cravate," passed over its middle, and its extremities are to be tied to the of the bed in a direction towards its foot; a similar bandage is to be placed on the anterior surface of the leg or the instep, and its ends fastened to the sides of the bed, in a direction towards its head. During the first month the thigh must be raised daily or nearly so, at the same time drawing it gently downwards, in order that both fragments may be in contact. When it is probable that consolidation has taken place the double-inclined plane is to be gradually lowered, by removing from time to time one of the pillows which form it, until they are all removed. The patients should keep their beds some days after this, and then should gradually commence walking, with great precautions. By employing this treatment, the fracture is consolidated without shortening, or at least with but little shortening, which is easily remedied by one heel being made rather higher than the other. Another advantage of this apparatus is, that it neither occasions pain nor uneasiness, which is of peculiar importance, when it is considered that old people are generally the subjects of this injury, in whom the work of reparation is peculiarly slow. Two cases are given of fracture of the neck of the thigh-bone; in the 1st, which occurred in a man 58 years of age, the pillows were removed about the 92d day after the injury, and no shortening had taken place—he could then move his legs with ease; on the 110th day he could walk easily with crutches. The 2nd case was that of a woman, aged 67; about the 65th day the apparatus was gradually removed—consolidation had taken place, and both limbs were of the same length. On the 85th day she left the hospital, cured, and using her limb very well.

### On Wounds of the Heart.

A few years since, all wounds of the heart were supposed to be mortal; but facts have proved that penetrating wounds, if narrow, are not only not instantaneously mortal, but that they may be cured. Thus, at Warsaw acupuncturation of the heart for cholera was practised. A ball was found lodged in the substance of the right ventricle of a soldier, who received the wound six years previously. Wounds of a few lines in extent, penetrating the cavities, are in some cases not so suddenly fatal as has been imagined.

*Case* 1. G. æt. 34, during a scuffle on Nov. 5th, 1831, received two wounds from a knife, one in the abdomen—the other between the fourth and fifth left ribs, on the edge of the cartilage of the fourth. From the wound in the chest there was considerable hemorrhage. He came into the Hôtel Dieu, his face pale and anxious, pulse extremely weak, but regular; pulsations of the heart regular, but hardly perceptible. The bleeding from the wound in the chest had ceased—there was no symptom of perforation of this cavity; left side sonorous, respiration regular, neither cough nor bloody expectoration. Nothing solid, liquid, or gazeous had escaped from the abdomen, which is supple. Patient is tranquil. The wounds were dressed with adhesive plaster.

6th. Pulse quicker; skin hot. Bleeding to 8 ozs.

7th. Same state; patient tranquil. Bleeding repeated. At night, there were symptoms of cerebral congestion, and he became affected with hemiplegia of the left side of the body; intellect uninjured.

8th. Same state. Cupping behind the ears.

9th. On auscultation, the following phenomenon was observed: when the chest was completely dilated, the air appeared to overcome an obstacle, and to rush into a cavity. The cerebral symptoms increased, and on the 12th he died.

*Examination—Abdomen.* A wound of two lines in length, penetrating into the cavity of the stomach; its edges were almost in contact, and partly agglutinated by mucus. The stomach was not inflamed, nor did it contain blood. *Thorax.* The cavity of the chest contained four ounces of blood, furnished by the wounded intercostal artery. There was a transverse wound, 3½ lines in length and one in breadth, of the middle part of the left ventricle, penetrating its cavity. The external fibres were the most separated, but the internal touched each other, thus closing the wound. The pericardium contained nearly an ounce of serum. *Brain.* Slight ramollissement of a part of the right hemisphere. It is probable that, in this case, the wound in the heart would have been cured, if the cerebral disease had not supervened.

*Case* 2. A large, sombre man, æt. 40, reduced to want by his misconduct, was imprisoned. He endeavoured to destroy himself by cutting off his penis with a knife, and although the instrument was blunt, he effected his purpose after using great force and perseverance. He was brought to the Hôtel Dieu, and ligatures were applied. For some days he remained in a calm state; but delirium, with other symptoms of cerebral excitement, came on, and he died in a semi-apoplectic state three weeks after the mutilation.

*Necropsy.* The membranes of the brain, as well as that organ were injected with blood. On raising the sternum, a large ecchymosis was seen on the pericardium, whose cavity was half filled with liquid blood. On seeking the source of this hemorrhage, many small wounds, filled by a fibrinous black clot, were discovered. In the centre of the ecchymosis of the pericardium, which occupied the anterior and superior part of the membrane, were two very narrow penetrating wounds, obliterated by small false membranes. On examining the outer part of the thorax, a small, round, cicatrized wound, about a line and a half in diameter, was found between the cartilages of the second and third rib of the left side. Beneath the skin, between the intercostals, and under the pleura was a large ecchymosis, extending from below upwards. The opening into the pleura was marked by a reddish-brown spot, and surrounded by pseudo membranes. The anterior border of the lung was not affected. There were five or six small wounds of the heart, the greater number penetrating the right ventricle. One was plunged into the interventricular septum—the other wounded, but did not penetrate the left ventricle. The substance of the heart was pale, and easily broken up by the fingers. The ventricles contained some black fibrinous clots. Chronic inflammation and ulceration of the gastro-intestinal, mucous membrane. On investigating the case, it was discovered that a long needle, used by saddlers, was taken from him in the prison after he had mutilated himself. This attempt at suicide must, therefore, have taken place twenty-five days before his death. This time also accords with the state of the external cicatrix. This very small and sharp instrument had been driven in vertically, and directed towards the heart; when its point had reached the organ, it had been pushed many times into its substance. The narrowness of the wounds had prevented any hemorrhage from the cavity of the ventricles, the few ounces of blood in the pericardium appearing to have come from the substance of the heart. The pulse of this patient, although often examined, presented no anomalies, nor were there any other symptoms of an injury of the centre of circulation.

*Case* 3. A man, æt 30, in a fit of jealousy, endeavoured to put an end to his existence, by inflicting five or six wounds on the præcordial region with a file, the end of which he had sharpened. He threw up blood from the mouth, and there was much hemorrhage from the wound. On being brought into the Hôtel Dieu, air was observed to escape from the wounds, indicating that they penetrated the chest. Respiration short and weak, pulse small and irregular, and so much debility that bleeding was impossible; when signs of re-action occurred he was bled, and the evacuation was repeated on every exacerbation. Thirty-six hours after the injury, the patient began to spit up thick expectoration, mixed with blood and pus. The wounds were then dressed, to prevent the introduction of air and hemorrhage; this seemed rather to relieve him. However, the symptoms became worse, and he died three days after the suicidal attempt. The fatal issue of the injury was hastened by the agitation produced by the sight of his mistress, and by an examination by the commissary of police.

*Necropsy*. There were two wounds above the left teat, and three below it. Considerable effusion of blood in the pleuræ; the left cavity contained air and black blood, partially coagulated, the quantity of which, together with the blood which escaped from the lungs, amounted to three or four pounds. Left lung compressed, and impermeable to air. Three or four openings in the left side of the pericardium, which contained a spoonful of blood and pus. Three wounds of the left ventricle, penetrating its cavity. On inflating the left lung, the air escaped at three openings, corresponding to the external wounds of the chest. The inner orifices of the wounds of the left ventricle were closed by small clots of blood. The blood which escaped externally, as well as that which was found in the pleura, came from a wounded intercostal artery.

M. Dupuytren has had many other similar wounds, in which the patients have lived two, three, four, five, six, eight, and fourteen days. In a thesis of M. A. Sanson, cases are given of wounds penetrating the left ventricle, where the patients have only lived five hours; and others, where both ventricles were injured, and life has been prolonged to five and twenty days. Such facts prove that penetrating wounds of the heart are not necessarily instantaneously mortal; they are undoubtedly very dangerous, but not hopeless.

*Symptoms.* If there are wounds of the præcordial region, with hemorrhage, general feebleness, faintness, small pulse, general pallor, cold extremities, vomiting, anxiety, want of sleep, oppression of the diaphragm, cold sweats, &c., there are great suspicions as to the nature of the injury. The diagnosis is difficult, as all these symptoms are rarely present. The treatment is similar to other penetrating wounds of the chest:—Bleeding, to diminish the quantity of blood and to prevent inflammation, according to the strength of the patient; repose; simple dressing to the wound, to prevent hemorrhage or the contact of air. If there is danger of immediate suffocation, from internal effusion of blood, the operation for empyema may be necessary; but this should never be attempted, unless the return of the heat and colour of the body indicate that internal hemorrhage has been arrested.

### On Lithotomy.

The hypogastric operation, or the incision of the bladder above the pubes, should never be had recourse to, unless the small space between the tuberosities of the ischium, the presence of tumours at the lower opening of the pelvis, or the large size of the stone, render its extraction beneath the pubes either difficult or impossible. Le Frère Côme operated on 84 persons of different age, sex, and health, and the mortality was one to four and a half, a much less favourable result than is obtained from a similar number of lateral operations. M. Dupuytren's mode of operation will be best explained by the following case. M. le R. æt. 62, of a middle size, strong constitution, sanguineous temperament, great activity, and a good liver, experienced for a long time difficulty in passing his urine, and subsequent pain. Together with these symptoms he has for 10 years been occasionally subject to hæmaturia, after strong exercise or excess. During two years these symptoms have been aggravated, flow of urine almost continual and involuntary; he was sounded, but the surgeon detected no stone, and treated him for catarrhus vesicæ. The disease however increased, the urine passed guttatim, causing great exertion and excessive pain. His urine was ammoniacal and appeared to be mixed with blood, pus, and mucus. He gradually emaciated and was confined to his bed. M. Dupuytren was consulted, and found, on sounding the patient, that the beak struck against the stone before it entered the cavity of the bladder. He with difficulty succeeded in passing the instrument partially between the stone and the bladder, and when there, it felt as in a vice: on withdrawing the sound, he passed his finger into the rectum, and found the lower part of the bladder filled, dilated, and hardened by the foreign body. On bending the trunk he explored the hypogastric region, and felt, behind the pubes, at the base of the median line of the abdomen, a hard, resisting and voluminous body, which he could raise with the indicator placed in the rectum. M. Dupuytren explained to the patient that the cause of his illness was a very large calculus, and the sufferer anxiously demanded its immediate removal. The next day M. Dupuytren with M. Sanson, and other assistants, performed the operation. The introduction of a silver catheter between the stone and the bladder was found to be impossible, an injection was then thrown in, but was immediately returned. M. Dupuytren then resolved to perform the operation above the pubes. Standing on the left side, he bent the patient's legs on his thighs, and these on the abdomen; M. Sanson, standing on the right side, introduced his finger into the rectum, and raised the stone so as to make it project as much as he could above the pubes. M. Dupuytren, with a bistoury, made a longitudinal incision three inches in length in the median line immediately above the pubes; he next divided the abundant adipose tissue, and the aponeurosis of the abdominal muscles; a transverse incision of a few lines was then made through the pyramidal muscles on each side, and between them the stone was felt more evident each time that M. Sanson raised it. Resting the cubital border of the indicator finger of the left hand on the symphysis pubis, and sliding on the angle of the finger the point of a sharp straight bistoury held in the other hand, M. Dupuytren plunged the instrument into the anterior part of the bladder immediately behind the symphysis pubis. White, thick, and consistent pus at the same moment filled the bottom of the wound, but as the knife had struck the stone this must have come from the bladder; the operator then enlarged the incision upwards for half an inch, and passing in the indicator finger which had guided the knife, he felt the stone, and ascertained that the bladder was half an inch in thickness; on further examination with the finger he found that there was a space of two inches between the most elevated point of the incision and the upper part of the bladder, which is generally covered by peritoneum; he therefore prolonged the incision upwards with a probe-pointed bistoury. The extraction was performed by means of the calculus forceps; having opened the branches and placed them one after the other over the stone, M. Dupuytren re-united them, and drawing them gradually upwards with a motion from right to left, whilst M. Sanson was also raising the calculus, he removed it. It was exactly the form of the bladder, which it had completely filled. It was 3½ inches in height, 3 inches in breadth, and 2½ inches in thickness. It was six ounces and a half in weight, and composed of the ammoniaco-magnesian phosphate. M. Dupuytren examined the bladder, which he found empty, and threw in an emollient injection which passed out with facility through the wound. The dressing was most simple; the patient was placed on his back, and his limbs slightly bent by a bolster placed beneath his hams, a large mesh of frayed linen was introduced between the lips of the wound into the bladder, over this was placed a piece of linen pierced with holes and covered with cerate, and above a layer of soft charpie. The abdomen was covered with flannel soaked with an emollient decoction. The patient felt immediate relief, and he slept two hours; in the night his belly became painful, with rigors followed by heat and fever; he was largely bled from the arm. In the morning he was considerably relieved, but as some tenderness remained 12 ounces of blood were taken; the urine flowed freely through the wound; on the fourth and fifth days suppuration was established, the urine soon began to pass through the urethra, and in about a month the cure was complete. Since this time the patient has enjoyed perfect health, with occasional symptoms of catarrhus vesicæ, which are readily removed. M. Dupuytren recommends, 1st, If the hypogastric operation is to be attempted, it should be done at first, and not as the last shift. 2dly. No incision should be made in the perinæum, but the sound (à dard) should be introduced immediately into the urethra, that is, when it is necessary to use it.

*Lateral Operation.* M. Dupuytren has been led, by experience and reflection on the frequently fatal termination of the common operation, to adopt a bi-lateral incision. In his first attempts at a new operation, he divided the neck of the bladder upwards towards the pubes, with the lithotome, after having made a longitudinal incision along the raphé. In a second operation he made a transverse incision with the lithotome; but although by the transverse division he avoided the two most formidable inconveniences of the lateral operation, wounding the rectum, or the arterial trunks, yet the bladder was reached with difficulty, and small calculi only could be extracted, whilst the rectum might be wounded as well as the scrotum by the longitudinal incision. Wishing therefore to preserve the advantages of the transverse incision, he made it semi-elliptic with its concavity backwards. Instead of making use of a common staff with an equal groove in its whole length, he used a lighter instrument, being of greater size, and having a deeper and wider groove at its great curvature for about two inches; the staff is terminated by an olivary protuberance, and is destitute of the cul-de-sac at the extremity of the common instrument. In order to divide the neck of the bladder, M. Dupuytren employs a double bistoire cachée, modified by M. Charrierè, an able instrument-maker of Paris. As this is to be obtained in all respectable surgical instrument-makers' shops we shall not enter into a description of it. The advantages of it are, that at a single stroke two openings can be made in the sides of the bladder, which vary in size from six to twenty lines in length.

*Operation.* The patient having been placed in the position adopted in the lateral operation, the channelled sound is introduced, and held perfectly vertically by an assistant. The operator rendering the integuments of the perinæum tense with his left hand, makes a semicircular incision with a double-edged knife, which commencing at the right, between the anus and the ischium, terminates at the left, at a corresponding point, having passed five lines in front of the anus, the anterior part of which it encircles. The instrument successively divides the subcutaneous cellular tissue, the superficial perineal aponeurosis, and the anterior point of the external sphincter ani. The commencement of the membranous portion of the urethra being exposed, the operator with the nail of the left indicator finger finds the groove of the sound, and directs the point of the knife into it. During the whole of the first part of the operation it is necessary to depress carefully the lower lip of the incision, to protect it, and to guard the rectum from a wound. After having made a small opening in the urethra, the lithotome held in the right hand, the thumb above and the two next fingers beneath it, is introduced into the groove of the sound, guided by the nail of the left indicator finger, which is held in the superior part of the wound. The contact of the two metallic bodies being well ascertained, the operator takes the handle of the catheter, and with his left hand depresses the instrument, so as to raise its point under the symphysis pubis, and slides the lithotome in the groove of the sound into the bladder. He then removes the catheter, and turns the lithotome, so that its concavity is towards the anus; then, grasping it with the right hand, he opens the two cutting instruments by compressing their extremities against the handle, and draws it out, not horizontally, but inclined downwards, until the blades are wholly withdrawn.

The left indicator finger being then introduced into the wound reaches the neck of the bladder easily, measures the extent of the opening, and guides the forceps. M. Dupuytren performed this operation twenty-three times in two years and a half, and but one case terminated fatally.

The following are the advantages of the bi-lateral operation. 1st. Its execution is more easy, expeditious, and sure, than the greater number of the other methods, particularly the lateral. 2d. The incision is made at the widest part of the pelvic outlet, and consequently at the most favourable point for the extraction of calculi. 3d. It is the shortest and most direct way to the bladder. 4th. It is superior to the lateral incision, as it makes an opening through the neck of the bladder and the prostate sufficiently large for the extraction of very voluminous calculi, without completely dividing the gland and making a dangerous incision. 5th. It avoids the ejaculatory canals. 6th. It may be practised on both sexes and at all ages. M. Dupuytren has performed this operation on about 70 individuals, six of whom have died, or one in twelve. He has operated with success on 26 persons successively. He does not pretend to assert that a fair average can be taken from so limited a number of cases, but when compared with a table which he has given of the results of other operations, the difference, even if a much greater proportion of fatal cases are allowed, is striking.

*TABLE of the Results of* 356 *Cases of Lithotomy collected during* 10 *Years, including those performed by M. Dupuytren as well as the most celebrated Surgeons of Paris or its Environs.*

| SEX. | AGE. | Number of Operations | Cures. | Deaths. | Proportion of Deaths to Cures. | |
|---|---|---|---|---|---|---|
| MALE.... | 3 to 15 years | 97 | 88 | 9 | 1 to 11 | 9 in 100 |
| | 15 to 30 ,, | 59 | 51 | 8 | 1 to 7 | 13½ ,, |
| | 30 to 50 ,, | 45 | 35 | 10 | 1 to 4 or 5 | 23 ,, |
| | 50 to 70 ,, | 74 | 56 | 18 | 1 to 4 | 24 ,, |
| | 70 to 90 ,, | 7 | 26 | 11 | 1 between 3 & 4 | 29½ ,, |
| FEMALE.. | 3 to 15 years | 7 | 7 | 0 | 0 — 0 | 0 in 100 |
| | 15 to 50 ,, | 11 | 10 | 1 | 1 to 3¾ | 10 ,, |
| | 50 to 70 ,, | 17 | 15 | 2 | 1 between 8 & 9 | 12 ,, |
| | 70 to 90 ,, | 9 | 7 | 2 | 1 between 3 & 4 | 22 ,, |
| TOTAL... | From 3 to 90.. | 356 | 295 | 61 | 1 to 6 | 17 in 100 |
| MEN..... | Ditto ........ | 312 | 256 | 56 | 1 between 5 & 6 | 18 in 100 |
| WOMEN.. | Ditto ........ | 44 | 39 | 5 | 1 to 9 | 11½ in 100 |

By this Table it appears, that in Paris the chances of death to cure after lithotomy are as one to five or six;—that the operation is best borne in infancy whilst the favourable chances diminish rapidly as the age increases. This mortality, which, if the different periods of time is considered, does not materially differ from that furnished by our provincial cases in the tables arranged by Mr. Smith, of the Bristol Infirmary, (Medico-Chirurgical Trans. Vol. XI. Part I.) proves lithotomy to be so dangerous an operation, that any modification of the common method which promises greater success must be hailed with pleasure. But so much depends on the skill of the operator, that a fair estimate cannot be made of the success of any particular operation even from a large number of cases if performed by the same surgeon. M. Dupuytren certainly states that he has lost but six out of seventy cases by this mode, but these are the last seventy operations he has performed, and there are two hospital surgeons in this metropolis, one of whom uses the *gorget*, and the other Blizard's knife, the average mortality of whose cases is considerably less. Indeed, if the success of one man is to be taken as a criterion of the advantages of one operation over another, the favourable results which followed the practice of the late Mr. Martineau of Norwich, in whose hands lithotomy could hardly be called dangerous, would be decisive of the advantages of the common uni-lateral incision.

Besides the articles we have analyzed, there is another on painful subcutaneous tumours, which have been so well described in this country by Mr. Wood, and which M. Dupuytren considers always to require extirpation; another on phlegmonous erysipelas, containing nothing very novel; and a third on artificial anus—a subject which M. Dupuytren has treated at length in another work, a full analysis of which was given in this Journal at the time. The concluding part of the second volume contains the substance of M. Dupuytren's Lectures on Gun-shot Wounds—the result of the experience afforded him by the sanguinary contests in Paris, particularly in July 1830, and June 1832, which filled the wards of the Hôtel Dieu with every species of injury of this description. To this subject we shall probably, at a future time, revert, in a short article.

